# Post-Transplant Sepsis After Liver Transplantation: Clinical Characteristics and Therapeutic Challenges

**DOI:** 10.3390/jcm15051989

**Published:** 2026-03-05

**Authors:** Vanja Silić, Nikolina Bašic-Jukić, Ivan Romić, Igor Petrović, Daniela Bandić Pavlović, Goran Pavlek, Emil Kinda

**Affiliations:** 1 Clinic for Anesthesiology, Reanimatology and Intensive Care, University Hospital Centre Zagreb, 10000 Zagreb, Croatiadaniela.bandic@mef.hr (D.B.P.); 2 Clinic for Internal Medicine, University Hospital Centre Zagreb, 10000 Zagreb, Croatia; nikolina.basic.jukic@kbc-zagreb.hr; 3 Department of Hepatobiliary and Transplantation Surgery, Clinic for Surgery, University Hospital Centre Zagreb, 10000 Zagreb, Croatia; igor.petrovic33@gmail.com (I.P.); goranpavlek@gmail.com (G.P.);

**Keywords:** sepsis, immunoparalysis, immunomodulation, liver, transplant

## Abstract

**Background:** Sepsis is one of the leading causes of early death after a liver transplant, with a frequency of up to 45% and a high death rate of 50% in more severe forms. Standard diagnostic and therapeutic algorithms are often not applicable to this specific population, where immunosuppression, reperfusion injury, and systemic inflammation overlap and generate a clinical picture that is significantly different from sepsis in immunocompetent patients. **Methods:** This paper analyzes the available literature and clinical experiences of characteristic immune and hemodynamic profiles of sepsis after liver transplants. Biomarkers (IL-6, IL-10, HLA-DR, lactate, and IgM) are discussed as tools for assessing immune status and guiding timely interventions, including the early application of continuous renal replacement therapy (CRRT) and the selective use of IgM-enriched immunoglobulins. **Results:** Sepsis after liver transplantation frequently unfolds in two phases, an initial hyper-inflammatory response driven by cytokine release and reperfusion injury and a second phase of secondary immunoparalysis characterized by reduced HLA-DR expression and increased anti-inflammatory signaling. The immunometabolic shift appears to influence the clinical course and may inform therapeutic decision-making. The immunoparalysis phase is accompanied by mitochondrial dysfunction and impaired vascular reactivity. This type of mechanism contributes to hemodynamic instability and a reduced response to standard therapy. Individualized monitoring and early use of hemofiltration and immunomodulatory measures can improve results in carefully selected patients. **Conclusions:** In this setting, an individualized immunometabolic approach may complement standard sepsis management in liver transplant recipients. The introduction of biomarkers of immune function into routine practice and the recognition of early signs of exhaustion of the immune response can assist in timely therapeutic decision-making and improve survival.

## 1. Introduction

Sepsis is one of the leading causes of death after a liver transplant. The frequency is between 25 and 45%, and mortality in severe forms can reach up to 50% [1,2,3]. Despite advances in surgical techniques, antimicrobial therapy, and perioperative care, infections and inflammation complications are still prevalent in the early post-transplant period [2,4,5].

The pathophysiology of sepsis in this context is complex and reflects the interplay of iatrogenic immunosuppression, ischemia–reperfusion injury, surgical factors, donor-related variables, and systemic inflammatory responses that may evolve in immunological paralysis [5,6,7]. Secondary immunodeficiency is commonly followed by endotoxemia and cytopathic hypoxia and can cause disturbances in oxidative phosphorylation and mitochondrial dysfunction despite adequate oxygenation [8,9,10,11,12,13]. The result is persistently elevated lactate levels, which more frequently reflect cellular metabolic collapse than only a circulatory problem [8,9,10,11,12,13,14,15,16,17].

Standard algorithms for diagnosing and treating sepsis were developed primarily for immunocompetent patients and often cannot be directly applied to transplant recipients [4,5,6]. Thresholds for initiating hemofiltration, interpretation of inflammatory markers, and response to therapy depend on the degree of immunosuppression and the patient’s metabolic status [14,15,16]. Hemodynamic instability in this population may reflect not only a consequence of infection but also varying degrees of immunological and metabolic dysregulation [13,18,19]. The potential role of early hemofiltration and the application of IgM-enriched immunoglobulins as supportive measures is considered an important component of supportive therapy [18,20,21].

The aim of this paper is to present a modern, pathophysiologically informed approach to sepsis after liver transplantation, with an emphasis on individualized immunomodulation and hemodynamic optimization, and integration of perioperative infectious risk factors into clinical decision-making. At the same time, these considerations do not intended to replace established infectious disease guidelines for solid organ transplantation. Current recommendations from The American Society of Transplantation Infectious Diseases Community of Practice comprehensively address donor-derived infections, perioperative antimicrobial strategies, and early post-transplant risk stratification. The present review seeks to complement these principles by adding a pathophysiological perspective to everyday clinical decision-making [22].

### Review Scope and Evidence Base

This narrative review was based on a topic search in the PubMed, MEDLINE and Embase databases. The keywords and combinations that were used included: “liver” and “post-transplant”; “transplant”; “sepsis”; “septic complications”; and “immunoparalysis”, “immunometabolism”, “extracorporeal support” and “immunomodulation”. All articles available up to the end of 2025 were considered. We only included articles that studied liver transplant and patients aged >18 and those that described septic complications. We prioritized articles that described both diagnostic and therapeutic approaches in the postoperative period. We synthetized conclusions from original studies as well as expert opinions in order to ensure pathophysiological context. The goal of this review was to place available evidence into the context of everyday clinical practice and to propose a phase-adapted conceptual framework for a better understanding and management treatment of sepsis after a liver transplant.

## 2. Discussion

### 2.1. Immunological Aspects of Post-Transplant Sepsis

The early post-transplant period is distinguished by dual immune dysfunction. Immunosuppressive therapy (calcineurin inhibitors, mycophenolates, and corticosteroids) is vital to prevent graft rejection and at the same time decrease the body’s ability to recognize and eliminate pathogens [2,5,7]. Conversely, the transplant itself results in reperfusion injury, which activates the massive release of pro-inflammatory cytokines (TNF-α, IL-6, and IL-1β), as well as activating neutrophils and complement, fostering a wave of systemic inflammation [6,11,13]. Along with quantitative changes in the immunological cellular population, post-transplant sepsis is also characterized by a pronounced qualitative dysfunction of the immune response. There is a disturbance of antigen presentation, reduction in phagocytic activity of macrophages, and weakened proliferative and cytotoxic response of T- lymphocytes. Disproportion between pathogen load and host immune response may contribute to the development of severe sepsis in liver transplant recipients, even in the absence of elevated body temperature or typical laboratory signs of inflammation [3,6,8]. Following the first pro-inflammatory wave, a phase of immunological paralysis usually occurs: exhaustion of the monocyte–macrophage system, a decrease in the HLA-DR expression on monocytes, and an increase in anti-inflammatory cytokines, especially IL-10 [6,23,24]. This shift in secondary immunodeficiency increases vulnerability to opportunistic and nosocomial infections, most commonly caused by Gram-negative bacteria (Enterobacteriaceae and Pseudomonas aeruginosa), enterococci, and fungi of the Candida genus [4,5,21].

**Figure 1:**
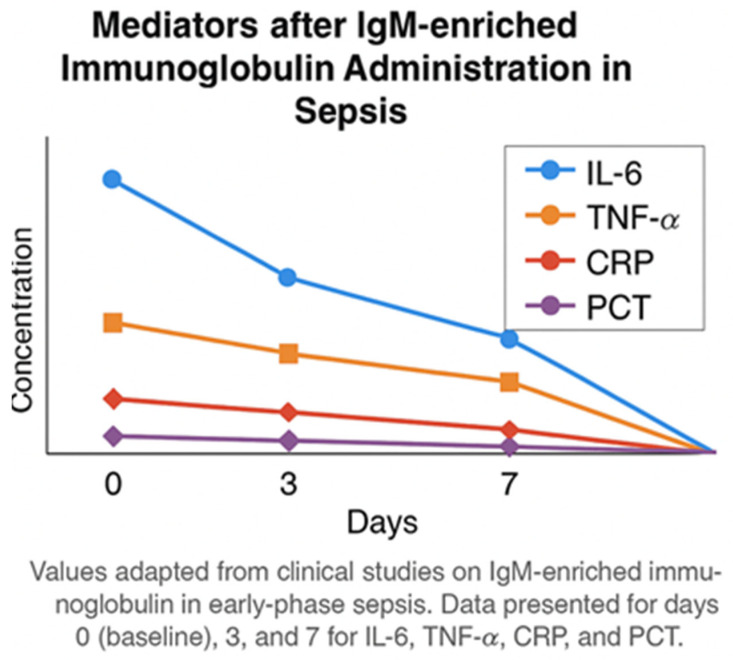
Biphasic immunometabolic trajectory of sepsis after liver transplantation. Abbreviations: IL—interleukin; TNF—tumor necrosis factor.

This evolving immune dysfunction can be conceptualized as a biphasic immunometabolic trajectory (Figure 1).

The simultaneous presence of iatrogenic immunosuppression and ischemia–reperfusion injury creates a unique immunological environment in which the initial hyper-inflammatory response rapidly turns into a state of immunological exhaustion. Reduced expression of HLA-DR on monocytes, persistent anti-inflammatory signaling and disturbed activation of innate immunity limit effective elimination of pathogens, with simultaneous maintenance of low-intensity systemic inflammation. In such circumstances in liver transplant recipients, especially in early post-transplant sepsis, functional IgM deficiency often develops. Such an immunometabolic profile may partly account for the atypical clinical preservation and limited response to standard anti-infective therapy observed in some liver transplant recipients. For these reasons, transplant recipients may benefit from individualized immunological assessment integrated with established infectious disease guidelines and perioperative risk stratification. Monitoring dynamic markers of immune function (e.g., monocyte HLA-DR, IL-6, and IL-10) may facilitate timely recognition of immunoparalysis phase and decision-making regarding targeted immunomodulatory strategies [18,20,21,23].

### 2.2. Hemodynamic and Metabolic Characteristics

The hemodynamic profile of sepsis in the early post-transplant period often differs from the classic picture of distributive shock. In the early post-transplant period, infection is frequently associated with metabolic and cellular changes that may contribute to hemodynamic instability [1,3,23]. After major surgical stress and reperfusion injury, the sensitivity of vascular receptors to catecholamines changes, vascular reactivity decreases, and relative resistance to vasopressors occurs. Mitochondrial dysfunction leads to a state of cytopathic hypoxia: cells cannot effectively use O_2_ despite adequate oxygen delivery. Elevated lactate then reflects a disturbance in cellular energetics, not necessarily hypoperfusion [3,8,24]. This hemodynamic–metabolic phenotype may, in part, reflect the immunometabolic disturbances described above, while also being influenced by surgical stress, donor characteristics, and perioperative management factors. Persistent endotoxemia, enhanced production of nitric oxide and mitochondrial dysfunction together disrupt microcirculation and the utilization of oxygen, creating disproportion between macrocirculatory parameters and actual tissue perfusion. Therefore, mean arterial pressure by itself does not necessarily reflect adequacy of oxygenation of the organs. Due to the combination of vasoplegia and impaired microcirculation, perfusion pressure (MAP) does not always support actual tissue oxygenation. The liver, as a newly transplanted organ, is particularly sensitive to fluctuations in portal and arterial flow, and small changes in systemic perfusion can cause functional ischemia of the graft [9,11]. This complicates decisions regarding volume resuscitation and vasopressor titration.

**Figure 2:**
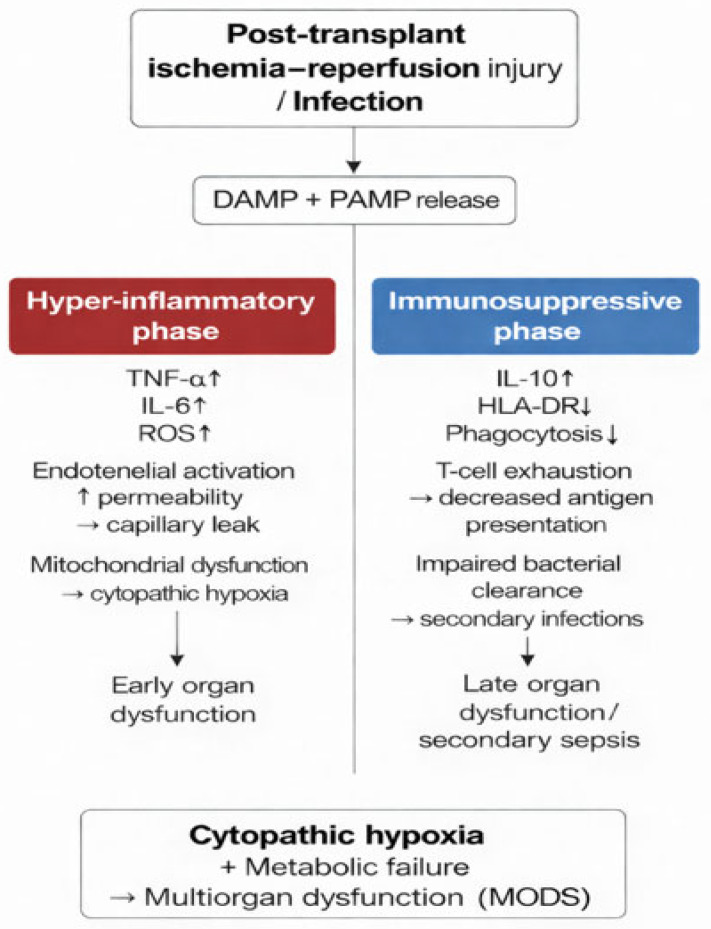
Biphasic immune response in post-transplant sepsis: from hyper-inflammation to immunosuppression. The arrow indicates temporal progression. Colors distinguish the different clinical phases: hyperinflammation (red), immunoparalysis (blue), and metabolic failure (gray); arrow represents temporal progression and that the colors correspond to different clinical phases (hyperinflammation, immunoparalysis, and metabolic failure).

In this context, an aggressive administration of volume directed exclusively at the normalization of lactate can be counterproductive, with potential worsening of graft congestion and an increase in intra-abdominal pressure and microcirculation. Therefore, trends of lactate should be interpreted within a broader metabolic and immunological context, together with established clinical and infectious disease management principles. Access to hemodynamic support must be individualized and multimodal. Advanced hemodynamic monitoring (PiCCO, EV1000, Swan-Ganz) is recommended with regard to assessing volume status, intrathoracic blood volume, and cardiac function [11,14]. Norepinephrine is the preferred therapy; vasopressin can be added in cases of catecholamine resistance [13,15]. Accordingly, biphasic immunological response and its hemodynamic–metabolic consequences suggest the management strategies may benefit from an integrated approach that considers immunological dysfunction, mitochondrial damage and circulatory instability, rather than focusing solely on isolated hemodynamic targets. The biphasic immune trajectory and its hemodynamic–metabolic consequences are illustrated in Figure 2.

From a clinical perspective, these hemodynamic and metabolic patterns should be observed in the context of liver transplants and should not be considered universally applicable to all forms of sepsis. These considerations are based on available evidence and clinical experience and are intended as a conceptual framework for understanding and not as formal therapeutic recommendations.

### 2.3. The Role of Hemofiltration and Hemoadsorption

Early continuous renal replacement therapy (CRRT) represents an important supportive component in the management of sepsis after a liver transplant, especially in acute kidney damage, metabolic acidosis, or fluid overload. However, its value goes beyond classic renal replacement therapy because it also acts in an immunomodulatory capacity by removing cytokines, inflammatory mediators, and endotoxins. In practice, the decision on CRRT is based on a combination of hemodynamic and metabolic indicators: persistent anuria or diuresis < 0.3 mL/kg/h for 6 h; lactate > 4 mmol/L despite optimized perfusion; MAP < 65 mmHg with noradrenaline ≥ 0.2 µg/kg/min; increase in SOFA score (>9). Early initiation within 12–24 h is associated with better stabilization of perfusion and reduced need for vasopressors. Hemoadsorbers (e.g., CytoSorb, Jafron HA-380, and Oxiris) have been developed to enhance the removal of cytokines and endotoxins. Although data in the transplant population are limited, the combination of CRRT and hemoadsorption may help stabilize hemodynamics and reduce IL-6/TNF-α. These procedures do not replace standard treatment of sepsis, which includes surgical source control, broad-spectrum antibiotics, and volume control. According to the center’s clinical experience, continuous venovenous hemofiltration (CVVH) or continuous venovenous hemodiafiltration (CVVHDF) is commonly used in clinical practice with controlled volume removal and caution to preserve graft perfusion. The choice depends on whether the aim is to employ diffusion to remove small molecules and uremic toxins or to focus only on convective clearance.

In the context of rising lactate, decreased urine output, and fluid retention, early hemofiltration may exert metabolic and immunomodulatory effects. In addition to removing toxins and maintaining volume, it can reduce circulating cytokines and stabilize the internal environment [10,14,20]. When initiated in appropriate clinical contexts, CRRT may reduce vasopressor requirements and support hemodynamic stabilization.

A variety of extracorporeal hemoadsorption and hemofiltration devices are currently available, each differing in adsorption capacity, target molecules, and biocompatibility with CRRT circuits. Table 1 summarizes the most commonly used systems and their specific characteristics in the context of liver transplant sepsis.

Early identification of immune exhaustion and metabolic failure may be supported by dynamic monitoring of circulating biomarkers. Table 2 presents key immune and metabolic markers that can help stratify patients and guide timing of immunomodulatory therapies.

It is important to recognize that extracorporeal blood purification techniques in post-transplant sepsis are used as additional and individually adapted therapeutic options. Because evidence in this population remains limited, decisions on their application should be based on clinical assessment and the immunological and metabolic status of the individual patient and not on predefined protocols. In available studies, early application of CRRT (most frequently within 24 h) has been associated with improved hemodynamic stabilization and reduced need for vasopressors, although survival data remain inconsistent. Contrarily, delayed application is often associated with persistent metabolic instability and a prolonged need for organ support. Data regarding IgM-enriched immunoglobulins suggest possible reduced mortality in selected patient pools with sepsis. At the same time, evidence specific to transplant patients remains restricted and is predominantly observational. The results of hemoadsorption strategies are heterogeneous, in relation to both reduction in cytokines and clinical outcomes, which likely reflects differences in timing of applications and patient selection. CRRT represents an important supportive intervention for acute kidney injury in the post-transplant period. Early CRRT may contribute to hemodynamic stabilization, while survival is generally lower for those needing long-term dialysis. But successful, prompt CRRT can bridge patients to recovery or successful transplant, with some studies showing high survival for specific, early-intervention cases.

We did not find studies that specifically compared outcomes of LT patients with sepsis between CRRT and IgM-enriched immunoglobulins. Several reports suggest that IgM-IVIG can be used as an adjunctive, immunomodulatory therapy in the perioperative management of liver transplant recipients, particularly those with high-risk infections or sepsis. Its use alongside CRRT aims to neutralize endotoxins and mitigate systemic inflammation. Overall, these therapies are better considered on a case-by-case basis rather than as routine or uniform practice [19,21].

### 2.4. IgM-Enriched Immunoglobulins (Pentaglobin): Selective Immunomodulation

Application of intravenous immunoglobulins in sepsis remains an area of interest and controversy, especially in patients with severe immune dysfunction. The IgM-enriched preparation (Pentaglobin) theoretically offers a wider spectrum of action than standard IVIGs thanks to a higher share of IgM and IgA, which partake in the neutralization of endotoxins and activation of complement [25,26]. Table 3 shows a comparison of IgM-enriched and standard IVIG preparation.

Mechanisms include binding bacterial toxins and superantigens, modulating receptor expression on monocytes and neutrophils, and promoting opsonization. This reduces the concentration of pro-inflammatory cytokines and mitigates the systemic inflammatory cascade, which may help “reset” the response in the immunoparalysis phase [27,28].

Clinical evidence for the effectiveness of Pentaglobin in the transplant population is limited and inconsistent. Studies in general sepsis show a potential reduction in mortality in selected groups with Gram-negative sepsis and high endotoxemia, but similar results have not been confirmed in transplant patients [25,28].

In summary, the available evidence supporting IgM-enriched intravenous immunoglobulins and extracorporeal cytokine adsorption in post-transplant sepsis remains limited and is largely based on observational studies. These interventions should therefore be considered as adjunctive, individualized strategies guided by immune and metabolic profiling, rather than as routine or standardized therapy. Their potential benefit appears to depend on timing, patient selection, and the underlying immune–metabolic phenotype.

Pentaglobin has a more favorable viscosity profile than standard IVIGs, but caution is needed in volume-overloaded or hemodynamically unstable patients. Slow infusion with continuous monitoring is recommended and, if necessary, simultaneous CRRT. Due to the high molecular weight, the preparation is not removed by CRRT, so dose adjustment is not required. Usage is considered selectively, especially in cases of proven Gram-negative bacteremia with prevalent endotoxemia, lower serum IgM (<0.4 g/L), or laboratory indicators of immunoparalysis (low HLA-DR; elevated IL-10) [26].The standard regimen is 250 mg/kg per day for three days.

Pentaglobin should not replace basic measures, surgical source control, antimicrobial therapy, and hemodynamic stabilization. Its application must be complementary and time-limited, with effect assessment within 48–72 h [26,28]. Pentaglobin has been recently investigated in LT in a study by Roat et al. The authors reported that in high-risk OLT recipients at high risk for infections, perioperative administration of an IgM-enriched preparation seems to reduce the development of new infections within the first 30 days after OLT [28].

**Figure 3:**
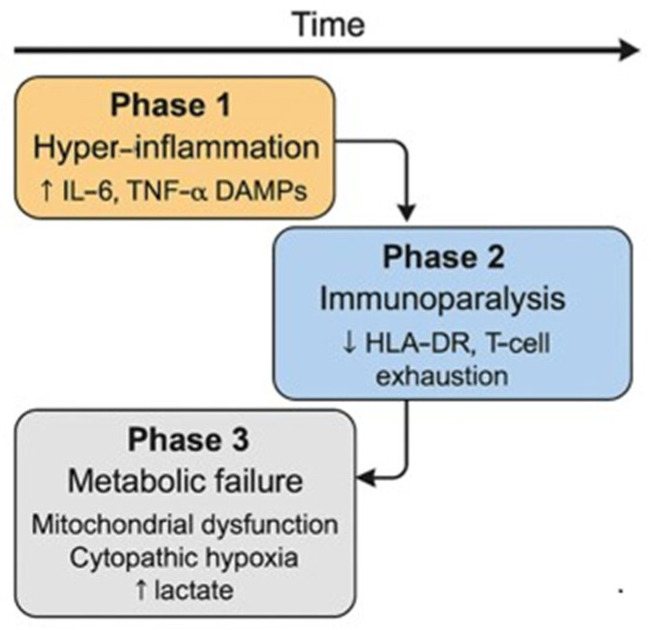
Dynamics of inflammatory markers. The arrow indicates the temporal progression of the immunological response.

The therapeutic effect of IgM-enriched intravenous immunoglobulins (IgM-IVIGs) can be visualized through their time-dependent impact on inflammatory mediators. As shown in Figure 3, levels of IL-6, TNF-αlfa, CRP and PCT progressively decline over the first week of therapy, reflecting attenuation of the systemic inflammatory response and improved immune balance.

Finally, as shown in Table 4, we developed a structured approach to post-transplant sepsis.

The proposed triggers are based on both published evidence and our center’s clinical experience. Key parameters in this table present our specific institutional guidelines and they are meant to guide structured clinical reasoning and so should not be interpreted as formal guideline-level thresholds. In everyday clinical practice, determination of biomarkers is most logical when the initial value is measured at the moment of suspicion of sepsis, followed by monitoring of repeated measures, most often every 24–48 h, depending on the clinical course of the patient. Independent single values may fluctuate, especially in immunocompromised transplant recipients.

## 3. Conclusions

Following a liver transplant, sepsis represents a special and extremely complex clinical problem, in which the classical understanding of it as an exclusively infection-triggered inflammatory reaction often does not fully explain the severity of the disease nor its course. In these patients, hemodynamic instability and organ failure do not arise only from pathogen burden but also from an associated disturbance of immunological regulation and cellular metabolism, which includes mitochondrial dysfunction, cytokine imbalance, endothelial damage and the development of secondary immunoparalysis. The clinical course of sepsis after a liver transplant often follows a biphasic pattern, with an initial hyper-inflammatory reaction which can relatively quickly transform into a state of immunological exhaustion. From this perspective, therapeutic approaches directed exclusively at macrocirculatory targets or escalation of antimicrobial therapy can be insufficient if, at the same time, deep immunological and metabolic dysfunction is not recognized and taken into account. This review approaches sepsis after a liver transplantation through the lens of immunometabolic dysregulation, while acknowledging its multifactorial clinical determinants. For this reason, an individualized and multidisciplinary approach to treatment is warranted. Early recognition of signs of immunological exhaustion, with a timely introduction of extracorporeal supportive methods such as continuous renal replacement therapy and selective hemoadsorption techniques can contribute to the stabilization of perfusion and mitigation of immunometabolic dysfunction. In carefully selected patients, application of IgM-enriched immunoglobulins can represent a targeted immunomodulatory option during the phase of immunoparalysis, under the condition of clear indications, an appropriate timing of administration and clearly defined therapeutic targets. Such an approach enables phase-adjusted therapeutic strategies which go beyond a routine escalation of antimicrobial therapy and focus exclusively on macrocirculatory parameters. In accordance with that, considerations presented in this work are intended to support clinical reasoning and individual decision-making and not to define strictly prescribed therapeutic algorithms.

## 4. Future Directions

Future research should be directed toward the development of reliable biomarkers, which would enable a more precise differentiation of phases of the immunological response after liver transplantation. The integration of immunological monitoring, including parameters such as IL-6, expression of monocytic HLA-DR and serum values of IgM, into clinical practice may represent an important step toward more individualized management of sepsis in this particularly vulnerable group of patients. Future studies should also consider how immunological biomarkers interact with donor-related factors and perioperative infectious management. A better understanding of these relationships may help refine risk assessment and support more individualized management of early post-transplant sepsis.

## Figures and Tables

**Table 1:** Hemoadsorption and hemofiltration systems used in sepsis after liver transplantation.

Oxiris	Jafron HA-380	CytoSorb	Feature
Combination: hemofiltration + hemoadsorption + anticoagulation	Hemoadsorption (resin cartridge with high surface area)	Hemoadsorption (porous polymer beads)	Mechanism of Action
IL-6, TNF-α, endotoxins, middle molecules	IL-6, TNF-α, cytokines (10–60 kDa)	IL-6, TNF-α, PCT, cytokines (10–60 kDa)	Target Molecules
Integrated into CRRT circuit (AN69 membrane)	Used with CRRT or standalone hemoperfusion	Compatible with CRRT, standalone setup	Compatibility with RRT
Also removes endotoxins, heparin-coated surface, integrated hemofilter	Cost-effective, high adsorptive capacity	Selective removal of cytokines, can be used in liver failure	Specific Features

**Table 2:** Biomarker-guided evaluation of sepsis progression in liver transplant recipients.

Suggested Thresholds/Interpretation	Typical Pattern After LT Sepsis	Pathophysiological Meaning	Biomarker
>100–200 pg/mL indicates systemic inflammation; >1000 pg/mL with shock	Rapid rise in hyper-inflammatory phase; may remain high with ongoing source	Early pro-inflammatory cytokine; correlates with severity	IL-6
High IL-10 with low IL-6 → functional immunoparalysis	Disproportionate rise vs IL-6 in later phase	Anti-inflammatory cytokine; marker of immune paralysis	IL-10
<8000–10,000 AB/c or <30% HLA-DR + monocytes = severe dysfunction	Decreased expression in immunoparalysis	Antigen-presentation capacity; global immune competence	Monocyte HLA-DR
<0.4 g/L supports IgM-IVIG candidacy	Low in prolonged sepsis or protein loss	First-line humoral defense; endotoxin neutralization	Serum IgM
>0.5–2.0 ng/mL = bacterial sepsis; failure to fall ≥ 80% at 72 h = poor response	Blunted rise under immunosuppression; trends useful	Bacterial infection marker; reflects activity	PCT
Trend > 50–100 mg/L supports inflammation	May be low early or on steroids	IL-6-driven acute-phase reactant	CRP
>4 mmol/L or <10% drop/6 h indicates severity	Persistent elevation in cytopathic hypoxia	Global perfusion and mitochondrial function marker	Lactate

**Table 3:** Comparison of IgM-enriched and standard IVIG preparation, including composition, immunological effects, efficacy in sepsis, dosing and clinical indications.

Standard IVIG	IgM-Enriched IVIG	Parameter
IgG > 95%	IgG (72%), IgA (12%), IgM (18%)	Composition
Primarily anti-inflammatory	Anti-inflammatory, anti-endotoxin, bactericidal	Immunological effects
No proven mortality benefit	Better in early phases (studies show lower mortality)	Effectiveness in sepsis
0.4 g/kg/day for 3–5 days	250–500 mg/kg/day for 3 days	Dosing
Primary immunodeficiencies, GBS, ITP	Sepsis, immunodeficiencies, transplant settings	Clinical indications

**Table 4:** Structured approach to post-transplant sepsis: clinical indicators, treatment objectives, and personalized immunological support.

Recommended Intervention	Therapeutic Target	Key parameters/Indicators	Clinical Phase
Immediate cultures, abdominal US/CT, initiate empiric broad-spectrum antibiotics	Early diagnosis and source identification	Mental status change, MAP < 65 mmHg, lactate > 2 mmol/L, urine output < 0.5 mL/kg/h, ↑CRP or PCT	Early recognition
Surgical/percutaneous drainage, anastomosis revision, targeted antimicrobial therapy	Eliminate infectious focus within 6 h	Bile leak, biloma, anastomotic failure, intra-abdominal collection	Source control
Noradrenaline (0.05–1 µg/kg/min), add vasopressin if catecholamine-resistant, restrictive fluids, PiCCO/EV1000 monitoring	Restore perfusion, maintain MAP ≥ 65 mmHg	Persistent hypotension, high vasopressor requirement, poor perfusion	Hemodynamic stabilization
Start CRRT within 12–24 h (low-flow continuous mode)	Metabolic stabilization and cytokine removal	Anuria > 6 h, lactate > 4 mmol/L, pH < 7.25, SOFA > 9	Early CRRT (CVVHDF)
Measure cytokines and immunoglobulins; evaluate timing for immunomodulation	Identify immune paralysis	HLA-DR ↓, IL-10 ↑, IgM ↓	Immune assessment
IgM-enriched IVIG (Pentaglobin 250 mg/kg/day × 3 days, slow infusion ± CRRT)	Support immune recovery	Gram-negative sepsis, endotoxemia, IgM < 0.4 g/L	Selective immunomodulation
Hemoadsorption (CytoSorb, Oxiris, hydrocortisone 200 mg/day	Reduce cytokine burden, improve perfusion	IL-6 > 1000 pg/mL, refractory hypotension, MODS	Adjunctive therapy
Repeat imaging, revise antibiotics, consider retransplantation	Reassess infection source and therapy	Persistent lactate elevation, no clinical improvement	Reevaluation (48–72 h)

**Abbreviations**: CRRT, continuous renal replacement therapy; CVVHDF, continuous veno-venous hemodiafiltration; MAP, mean arterial pressure; MODS multiple organ dysfunction syndrome. **↑** indicates increased levels; **↓** indicates decreased levels.

## Data Availability

No new data were created or analyzed in this study. Data sharing is not applicable to this article.
